# Identification of novel mobile colistin resistance gene *mcr-10*

**DOI:** 10.1080/22221751.2020.1732231

**Published:** 2020-03-02

**Authors:** Chengcheng Wang, Yu Feng, Lina Liu, Li Wei, Mei Kang, Zhiyong Zong

**Affiliations:** a Center of Infectious Diseases, West China Hospital, Sichuan University, Chengdu, People’s Republic of China; b Division of Infectious Diseases, State Key Laboratory of Biotherapy, Chengdu, People’s Republic of China; c Center for Pathogen Research, West China Hospital, Sichuan University, Chengdu, People’s Republic of China; d Department of Infection Control, West China Hospital, Sichuan University, Chengdu, People’s Republic of China; e Laboratory of Clinical Microbiology, Department of Laboratory Medicine, West China Hospital, Sichuan University, Chengdu, People’s Republic of China

**Keywords:** Colistin resistance, *mcr*, *mcr-10*, plasmid, *Enterobacter roggenkampii*

## Abstract

Mobile colistin resistance (*mcr*) genes represent an emerging challenge. Here we describe a novel *mcr* gene, *mcr-10*, on an IncFIA plasmid of an *Enterobacter roggenkampii* clinical strain. *mcr-10* has the highest nucleotide identity (79.69%) with *mcr-9* and encodes MCR-10 with 82.93% amino acids identical to MCR-9. *mcr-10* confers 4-fold increase in colistin MIC (from 1 to 4 mg/L) when cloned into a colistin-susceptible *E. roggenkampii* strain. By screening GenBank, *mcr-10* was found in various *Enterobacteriaceae* species of countries in four continents, suggesting that this gene has widely spread. MCR-10 shows 79.04% to 83.67% amino acid identity and highly conserved predicted protein structures with chromosomally encoded MCR-like phosphoethanolamine transferases (designated MCR-B here) of various *Buttiauxella* species. MCR-10, MCR-9 and MCR-B proteins may, therefore, originate from a common ancestor. *mcr-10* was adjacent to a site-specific recombinase-encoding gene and was bracketed by IS*903* and may be mobilized by site-specific recombination or composite transposon. Our results indicate that *mcr-10* is a novel plasmid-borne colistin resistance gene and warrants immediate monitoring and further studies.

## Introduction

Colistin is a last resort antimicrobial agent against carbapenem-resistant Gram-negative bacteria including *Enterobacteriaceae* but strains with acquired colistin resistance have also emerged worldwide [1]. Colistin resistance in the *Enterobacteriaceae* can be due to chromosomal mechanisms and plasmid-borne mobile colistin resistance genes (*mcr*). Since the report of the first *mcr* gene, *mcr-1*, in 2016 [2], a few other *mcr* genes including *mcr-2* [3], *mcr-3* [4], *mcr-4* [5], *mcr-5* [6], *mcr-6* [7], *mcr-7* [8], *mcr-8* [9] and *mcr-9* [10] have been described in *Enterobacteriaceae* and *mcr-1* and *mcr-4* have also been reported in *Acinetobacter* spp.[11,12] or *Pseudomonas* spp. (*mcr-1* only) [12]. All of the MCR proteins are phosphoethanolamine (PEA) transferases [13]. These PEA transferases catalyse the attachment of PEA to lipopolysaccharides (LPS)-lipid A, lead to a reduction of the negative charge of LPS upon structural alteration of lipid A and therefore result in resistance to colistin [13]. Of note, the discovery of *mcr-9* in colistin-susceptible strains suggests that strains carrying *mcr* genes may not exhibit colistin resistance phenotype due to low-level gene expression [10]. This promotes us to investigate the presence of *mcr-*like genes in a colistin-susceptible *Enterobacter* clinical strain, for which the minimum inhibitory concentration (MIC) of colistin was 2 mg/L, close to the 4 mg/L resistance breakpoint defined by the European Committee on Antimicrobial Susceptibility Testing (EUCAST) (http://www.eucast.org/). We found a new *mcr* variant, designated *mcr-10*, in the strain and report the findings here.

## Methods

### The strain, *in vitro* susceptibility testing

Strain 090065 (also called WCHER090065) was a clinical isolate recovered in 2016 at West China Hospital. This study was approved by the Ethical Committee of West China Hospital with waiving the inform consent. MICs of aztreonam, cefepime, ceftazidime, colistin, imipenem, meropenem, piperacillin-tazobactam, and tigecycline were determined using the broth microdilution method of the Clinical and Laboratory Standards Institute (CLSI) [14]. For colistin and tigecycline, the breakpoints defined by EUCAST (http://www.eucast.org/) were applied.

### Genome sequencing and analysis

Strain 090065 was subjected to whole-genome sequencing using the HiSeq X10 (Illumina; San Diego, CA, USA) according to the manufacturer’s instructions. Genomic DNA was prepared using the QIAamp DNA Mini Kit (Qiagen, Hilden, Germany). Generated reads were *de novo* assembled into contigs using SPAdes v3.13.0 [15] applying the careful and auto-cut-off modes. To determine the location of *mcr-10*, strain 090065 was also subjected to long-read whole-genome sequencing using a MinION Sequencer (Nanopore; Oxford, UK). The *de novo* hybrid assembly of both short (Illumina) and long reads was performed using Unicycler v0.4.3 [16] under conservative mode for increased accuracy. Pilon v1.22 [17] was used to correct complete circular contigs with Illumina reads for several rounds until no change was detected.

Prokka v1.13 [18] was used to annotate the genome sequence. Antimicrobial resistance genes were identified from genome sequences using the ABRicate program (https://github.com/tseemann/abricate) to query the ResFinder database (https://cge.cbs.dtu.dk/services/ResFinder/). Plasmid replicons were identified using PlasmidFinder 2.0 (https://cge.cbs.dtu.dk/services/PlasmidFinder/).

#### Nucleotide sequence accession numbers

The complete sequence of the chromosome and plasmids of strain 090065 has been deposited into GenBank under the accession no. CP045064-CP045066. The sequence of *mcr-10* has been deposited into GenBank under the accession no. MN179494.

### Precise species identification

For precise species identification, the pair-wise average nucleotide identity (ANI) between strain 090065 and type strains of *Enterobacter* species was determined using JSpeciesWS based on BLAST [19]. A ≥ 95–96% ANI cut-off was used to define a bacterial species [20].

### Cloning

The −10, −35 boxes of the promoter of *mcr-10* were predicted using the online tool BPROM (http://www.softberry.com/berry.phtml?topic=bprom&group=programs&subgroup=gfindb). The complete coding sequence of *mcr-10* and its promoter region were amplified with primers 090065_up_SacI (5′-AAAAAAGAGCTCTCCGCTTTGTATCCCAATAC; restriction site is underlined) and 090065_down_EcoRI (5′-AAAAAAGAATTCTTTTATAATTTCCGGCAGCA) using PrimeSTAR Max DNA Polymerase (Takara, Dalian, China). PCR amplicons and the vector pBC SK (Stratagene, La Jolla, CA, USA) were digested using *Sac*I and *EcoR*I (New England Biolabs, Ipswich, MA, USA) and were ligated to the pBC SK vector using T4 ligase (New England Biolabs) to construct pBC SK-mcr10. pBC SK-mcr10 was transformed into a colistin-susceptible *E. roggenkampii* clinical strain, named 120033, by chemical transformation. Potential transformants containing *mcr-10* were selected on Luria–Bertani agar plates (Sigma; St. Louis, MO, USA) containing 30 mg/L chloramphenicol. Colonies on plates were screened for *mcr-10* by PCR using primers 090065_up_SacI/090065_down_EcoRI and the presence of *mcr-10* was confirmed by Sanger sequencing amplicons. MICs of colistin were determined for transformants containing pBC SK-mcr10 using the CLSI broth microdilution method.

### Induction tests

To examine whether the expression of *mcr-10* is inducible as reported for *mcr-9* [10], strain 090065 was subjected to induction with IPTG (isopropyl-β-d-thiogalactopyranoside; BBI, Shanghai, China) or lactose (Meilun, Dalian, China) as described previously [21]. After induction, MIC of colistin for strain 090065 was determined in the absence or presence of 1 or 3 mmol/L lactose or 0.4 or 1 mmol/L IPTG.

### Conjugation and electroporation experiments

Conjugation experiments were carried out in broth and on filters with the azide-resistant *E. roggenkampii* strain 120033 AizR (an azide-resistant variant of 120033) as the recipient at both 25°C and 37°C as described previously [22]. Potential transconjugants were selected on LB agar plates containing 2 mg/L colistin and 150 mg/L azide. Electroporation was performed with *Escherichia coli* DH5α as described previously [23]*.* Transformants were selected on Luria–Bertani agar plates containing 2 mg/L colistin. The presence of *mcr-10* in the transformant was confirmed by PCR with primers 090065_up_SacI/090065_down_EcoRI and subsequent Sanger sequencing. MICs of aztreonam, ceftazidime, colistin, and meropenem were determined as described above.

### Screening the presence of *mcr-10* in GenBank

We screened the presence of *mcr-10* in sequences including complete or draft genome sequences deposited in GenBank by BLAST (https://blast.ncbi.nlm.nih.gov/Blast.cgi, accessed by 30 August 2019). Matches with >90% identity and >90% coverage were retrieved from GenBank.

### Structure comparisons of MCR-10, MCR-9 and MCR-Bs

The amino acid sequences of MCRs were retrieved from Bacterial Antimicrobial Resistance Reference Gene Database (BioProject no. PRJNA313047). MCR-B proteins from genus *Buttiauxella* were retrieved from their whole-genome assemblies. Along with the two alleles of MCR-10 found in this study (see below for details), the amino acid sequences of all genes (*n* = 75) were aligned using Prank v1.70427 [24] with 50 iterations first, followed by aligning corresponding nucleotide sequences using aligned amino acid sequences as the guide in the same program. The aligned nucleotide and amino acid sequences were fed into RAxML v8.2.12 [25] with a 1000-bootstrap test under GTRGAMMA and PROTGTRGAMMA model, respectively, for inferring maximum-likelihood phylogenies.

Three-dimensional (3D) structural models of MCR-10, other reported MCR proteins (MCR-1 to -9) and MCR-B of various *Buttiauxella* species based on lipid A PEA transferase [26] were constructed using Phyre2 [27] and were visualized using UCSF Chimera [28]. Secondary structures of MCR-9, MCR-10 and MCR-Bs were then predicted using ESPript 3 [29].

## Results

### A novel *mcr*, *mcr-10*, was identified in strain 090065 of *E. roggenkampii*

Strain 090065 (also called WCHER090065) was recovered from an ascites sample of a patient in 2016 at West China Hospital. Strain 090065 (also called WCHER090065) was resistant to aztreonam (MIC, 256 mg/L), ceftazidime (MIC, 64 mg/L), imipenem (MIC, 32 mg/L), meropenem (MIC, 16 mg/L), intermediate to piperacillin-tazobactam (MIC, 32/4 mg/L) but susceptible to cefepime (MIC, 2 mg/L), colistin (MIC, 2 mg/L), and tigecycline (MIC, 2 mg/L). Short-read whole-genome sequencing of strain 090065 generated 1.77 clean gigabases (367.6× coverage), which were *de novo* assembled into 137 contigs (*N50*, 134,938 bp). Strain 090065 belongs to *E. roggenkampii*, a recently described *Enterobacter* species [30] as it has 98.51% ANI value with the type strain of *E. roggenkampii* (strain DSM16690^T^, GenBank accession no. CP017184).

Strain 090065 has two known antimicrobial resistance genes, *bla*_MIR-5_ (a chromosomal *ampC* gene intrinsic to *Enterobacter* mediating resistance to aztreonam, 1st to 3rd cephalosporins, and penicillins) and *fosA* (mediating resistance to fosfomycin). Known *mcr* genes including *mcr-1* to *-9* were not identified in the draft genome sequence of strain 090065. However, a PEA transferase-encoding gene, which has 79.69% identity and 99% coverage with *mcr-9.1* (GenBank accession no. NG_064792) [10], was identified. The PEA transferase encoded by the gene has 82.93% amino acid identity with MCR-9, suggesting that the transferase is a novel MCR-like protein.

To determine whether this novel *mcr-*like gene mediates colistin resistance or not, the gene was cloned on pBC SK (Stratagene, La Jolla, CA, USA) to construct pBC SK-mcr10, which was transferred into a colistin-susceptible *E. roggenkampii* clinical strain, named 120033. MIC of colistin against strain 120033 containing pBC SK-mcr10 and strain 120033 containing pBC SK alone was 4 and 1 mg/L, respectively. The four-fold increase in colistin MIC in the presence of *mcr-10* suggests that this gene indeed mediates colistin resistance.

Although the precise cut-off to define MCR groups has been established, the amino acid identity of 88% to 96% has been commonly used the *de facto* cut-off [31]. After consulting the NCBI as suggested recently [31], the novel MCR identified in the present study is named MCR-10. MCR-10 has 29.31%, 27.09%, 61.60%, 42.49%, 28.94%, 26.53%, 58.26%, 35.81%, and 82.93% amino acid identity with MCR-1, MCR-2, MCR-3, MCR-4, MCR-5, MCR-6, MCR-7, MCR-8, and MCR-9, respectively. Induction with lactose and IPTG did not increase the MICs of colistin against 090065 as MIC of colistin remained 2 mg/L in the presence of 1 or 3 mmol/L lactose or 0.4 or 1 mmol/L IPTG. This suggests that the expression of *mcr-10* may not be inducible.

### *mcr-10* was carried by an IncFIA plasmid in strain 090065

To determine the location of *mcr-10*, strain 090065 was also subjected to long-read whole-genome sequencing using MinION. The hybrid assemblies of Illumina and MinION reads revealed that strain 090065 had a 4.86-Mb circular chromosome and two plasmids, p1_090065 (10,944-bp, replicon type undetermined) and pMCR10_090065 (71,775-bp, containing an IncFIA(HI1) replicon).

Despite repeated attempts of conjugation experiments, transconjugants containing pMCR10_090065 were not obtained, suggesting that pMCR10_090065 was not self-transmissible. Examining the complete sequence of pMCR10_090065 revealed that there was no conjugation module on this plasmid. *E. coli* transformant carrying *mcr-10* was obtained by electroporation, confirming that *mcr-10* is plasmid-borne. *mcr-10*-carrying transformant was susceptible to colistin (MIC, 2 mg/L), meropenem (MIC, <1 mg/L), aztreonam (MIC, <1 mg/L), and ceftazidime (MIC, 4 mg/L). This suggests that the resistance to these agents seen in strain 090065 was not co-transferred with *mcr-10*.

### *mcr-10* is found in a few genera of the family *Enterobacteriaceae* and has a global distribution

In GenBank, a total of 34 matches that have >90% identity and >90% coverage with *mcr-10* were identified including 30 draft genome sequences (Table 1) and four complete plasmid sequences (Table 2). In addition, an incomplete *mcr-10* was found in the draft genome sequence of an *E. coli* strain (accession no. LLYM01000000), which was truncated by insertion sequence IS*3*. The *mcr-10*-containing strains belonged to 13 species of 6 genera (*Citrobacter*, *Enterobacter*, *Escherichia*, *Klebsiella*, *Kluyvera*, and *Raoultella*) of the family *Enterobacteriaceae* but most (24/35) were of the genus *Enterobacter*. The strains were found in 11 countries (Australia, Canada, China, France, Germany, Japan, the Netherlands, Spain, Thailand, USA and Vietnam) of four continents. Most (*n* = 30) of the 35 strains were from human, *mcr-10* was also present in strains from animal (dog, *n* = 1) and environment (water, *n* = 4). As only contigs of the 31 draft genome sequences are available, we were unable to reliably determine the location (chromosome or plasmid) of *mcr-10* for these strains. Nonetheless, all of the four *mcr-10*-carrying plasmids contained one or two IncF replicons (Table 2).

**Table 1.** Strains harbouring *mcr-10* in GenBank.

Host Species	Strain	Accession no.	Country	Year	Host	Source
*Citrobacter freundii*	B38^a^	GCF_001702455	China	1998	Human	Leg ulcer
*Enterobacter asburiae*	KA2	GCF_003023805	Spain	2014	Human	Rectal colonization
*Enterobacter cloacae*	SB610	GCA_900978275	Netherlands	2000	Environment	Water
*Enterobacter cloacae*	PIMB10EC27^a^	GCF_002982195	Vietnam	2010	Human	Urine
*Enterobacter kobei*	C7	GCF_001276465	Australia	2003	Human	Lungs
*Enterobacter kobei*	24.1-R2	GCF_002001845	Australia	2012	Human	Feces
*Enterobacter kobei*	4300STDY7045912	GCA_900496815	Thailand	2016	Human	NA
*Enterobacter kobei*	GEO_33_Down_A	GCF_004024245	USA	2017	Environment	Water
*Enterobacter kobei*	GEO_23_Down_A	GCF_004024335	USA	2017	Environment	Water
*Enterobacter kobei*	MGH132	GCF_002151855	USA	2015	Human	NA
*Enterobacter kobei*	UCI 39	GCF_000534155	USA	NA	Human	Urine
*Enterobacter kobei*	GN02570	GCF_001022655	USA	2007	Human	Bodily fluid
*Enterobacter kobei*	1001_ECLO	GCF_001052605	USA	NA	Human	NA
*Enterobacter kobei*	1000_ECLO	GCF_001052055	USA	NA	Human	NA
*Enterobacter kobei*	SMART_313	GCF_001472135	Vietnam	2010	Human	NA
*Enterobacter roggenkampii*	49530189	GCF_002208285	Australia	2017	Human	Blood
*Enterobacter roggenkampii*	ECC1097	GCF_002785795	China	2010	Human	Urine
*Enterobacter roggenkampii*	GER_MD16_1505_Eko_090	GCF_003331015	Germany	2015	Dog	Feces
*Enterobacter roggenkampii*	ntmc-TH	GCF_003427235	NA	2017	Human	Blood
*Enterobacter roggenkampii*	MGH 25	GCF_000492995	NA	NA	Human	Urine
*Enterobacter roggenkampii*	GN05753	GCF_001518455	USA	2013	Human	NA
*Enterobacter roggenkampii*	GN02204	GCF_001023195	USA	2003	Human	Body fluid
*Enterobacter roggenkampii*	GN02825	GCF_001022915	USA	2009	Human	Body fluid
*Enterobacter sichuanensis*	ECC1752	GCF_002785945	China	NA	Human	NA
*Enterobacter* sp.	18A13^a,b^	AP019634	Japan	2018	Environment	River water
*Escherichia coli*	AZ74^c^	GCF_001484485	China	2014	Human	Gut
*Escherichia coli*	CRE54	GCF_003401245	USA	2016	Human	Blood
*Klebsiella pneumoniae*	1323_ECLO	GCF_001053395	USA	NA	Human	NA
*Klebsiella pneumoniae*	BIDMC 67	GCF_000692255	USA	2013	Human	Abscess
*Klebsiella quasipneumoniae*	149G8	GCF_003289115	France	2017	Human	Urine
*Klebsiella quasipneumoniae*	CRE71	GCF_003401175	USA	2016	Human	Blood
*Kluyvera* sp.	TUM14004	GCF_004310925	Japan	2013	Human	Blood
*Raoultella electrica*	TUM14061	GCF_004312065	Japan	2013	Human	Blood
*Raoultella ornithinolytica*	FDAARGOS_431^a^	GCF_002635365	Canada	2015	Human	Rectal swab

Note*:* NA, not available.

^a^In the four strains, *mcr-10* is located on a plasmid (Table 2).

^b^*mcr-10* in strain 18A13 encodes an MCR-10 variant with 15 amino acids different from MCR-10 encoded by *mcr-10* genes in all other strains.

^c^*mcr-10* in strain AZ74 is truncated.

**Table 2.** *mcr-10-*carrying plasmids.

Plasmid	Accession no.	Species, strain	Source	Year	Country	Plasmid replicon
pMCR10_090065		*Enterobacter roggenkampii* 090065	Human ascites	2016	China	FIA
pOZ172	CP016763	*Citrobacter freundii* B38	Human leg ulcer	1998	China	FIB, FII
pEC27-2	CP020091	*Enterobacter cloacae* PIMB10EC27	Human urine	2010	Vietnam	FII
unnamed1	CP023893	*Raoultella ornithinolytica* FDAARGOS_431	Human rectal swab	2015	Canada	FIB, FII
pECC18A13-1^a^	AP019635	*Enterobacter* spp. 18A13^b^	River water	2018	Japan	FIA

^a^The MCR-10 variant encoded by pECC18A13-1 has 15 amino acid substitutions compared with MCR-10 encoded by the other plasmids.

^b^The strain is likely of a new *Enterobacter* species, which is most closely related to *E. roggenkampii* with a 94.51% ANI value.

The *mcr-10* genes of 33 strains encode MCR-10 that has an identical amino acid sequence with that in strain 090065. The remaining sequence of the truncated *mcr-10* (accession no. LLYM01000000) is also identical to that of strain 090065. However, another MCR-10 variant with 15 amino acid substitutions (97.2% [524/539] amino acid identity) is found in plasmid pECC18A13-1 of *Enterobacter* sp. 18A13 (all call as DSM16690; GenBank accession no. AP019635). The alignment of the two MCR-10 variants is shown in Figure S1 in the Supplementary file. This suggests that *mcr-10* has been diverged.

### *mcr-10* may originate from a yet-to-identified species closely related to known *Buttiauxella* species

Like MCR-9 [10,32], MCR-10 shows significant amino acid identity with chromosomally encoded MCR-like PEA transferases of various *Buttiauxella* species, designated MCR-B here, from 79.04% (426/539 identical aa) with that of *Buttiauxella agrestis* (NCBI Reference Sequence no. WP_034495833.1) to 83.67% (451/539 identical aa) with those of *Buttiauxella gaviniae* (NCBI Reference Sequence no. WP_064511805.1) and *Buttiauxella brennerae* (NCBI Reference Sequence no. WP_064558897.1).

**Figure 1.**
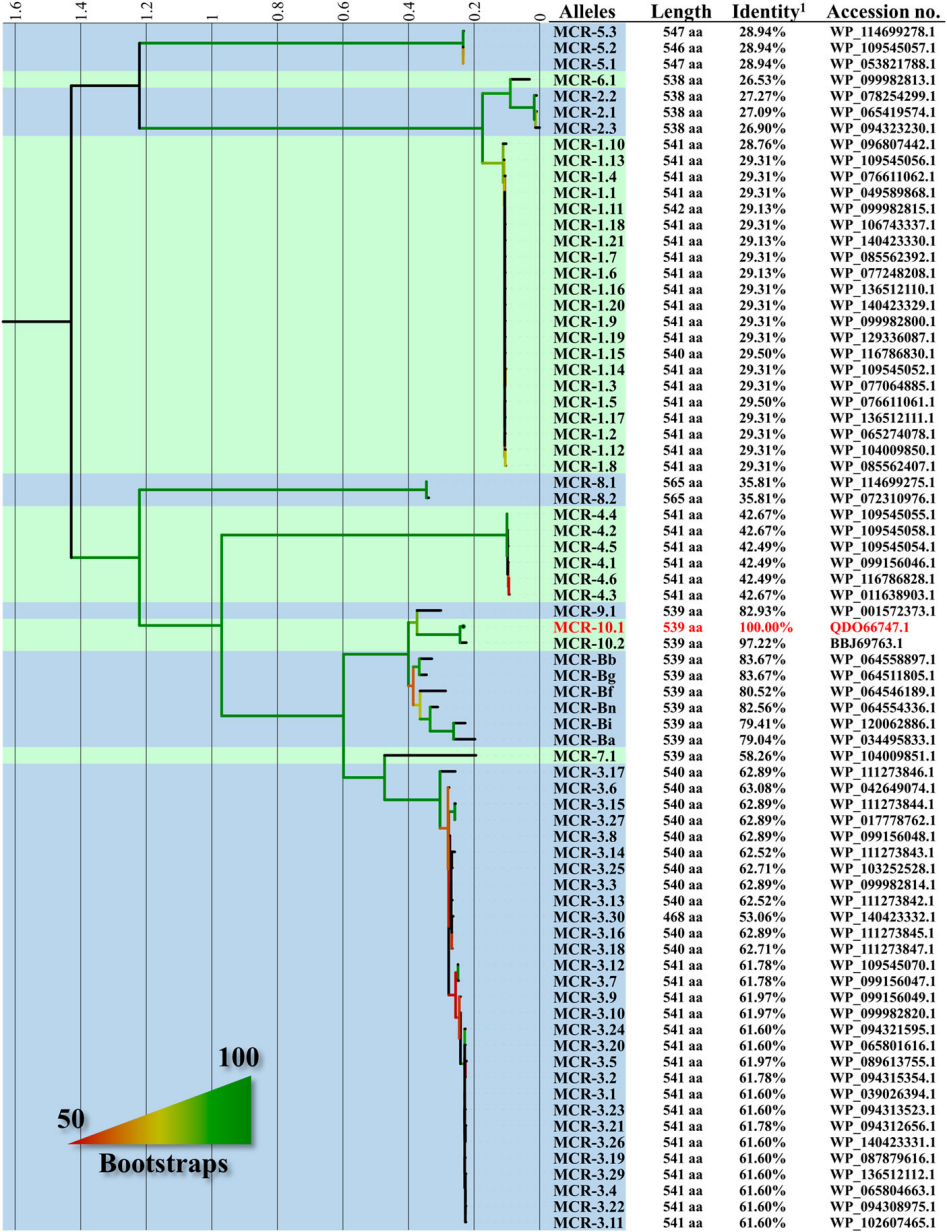
Comparison of MCR-10 with other known MCRs and MCR-like proteins (MCR-B) in *Buttiauxella* species. This maximum-likelihood tree based on amino acid sequences was inferred using RAxML v8.2.12 [25] with a 1000-bootstrap test under the PROTGTRGAMMA model. The tree is middle-point rooted and the blue and green strips separate different MCR families with MCR-10 being highlighted in red. Bootstrap results are indicated by colour gradient on the branches, starting from 50% shown as red and up to 100% shown as green. MCR-like proteins in *Buttiauxella* species are named here according to the species. MCR-Ba, MCR-Bb, MCR-Bf, MCR-Bg, MCR-Bi, and MCR-Bn are MCR-like proteins from *Buttiauxella agrestis* strain ATCC 33320^T^ (accession no. JMPI00000000), *Buttiauxella brennerae* ATCC 51605^T^ (accession no. LXER00000000), *Buttiauxella ferragutiae* ATCC 51602^T^ (accession no. LXEQ00000000), *Buttiauxella gaviniae* ATCC 51604^T^ (accession no. LXEP00000000), *Buttiauxella izardii* CCUG35510^T^ (accession no. QZWH01000000), and *Buttiauxella noackiae* ATCC 51607^T^ (accession no. LXEO00000000), respectively.

Comparison with known MCRs (MCR-1 to MCR-9) and MCR-B of various *Buttiauxella* species showed MCR-10 form a cluster with MCR-9 and MCR-B, which is well separated from other MCRs (Figure 1; a phylogenetic tree of *mcr* genes is shown as Figure S2 in the Supplementary file). Nonetheless, there are a large number of amino acid variations (≥89 aa) between MCR-10 and MCR-B proteins and the variations are diffusely distributed in the amino acid sequence of the MCR-B proteins (Figure S3 in the Supplementary file).

### MCR-10, MCR-9 and MCR-Bs are highly similar at the structural level

**Figure 2.**
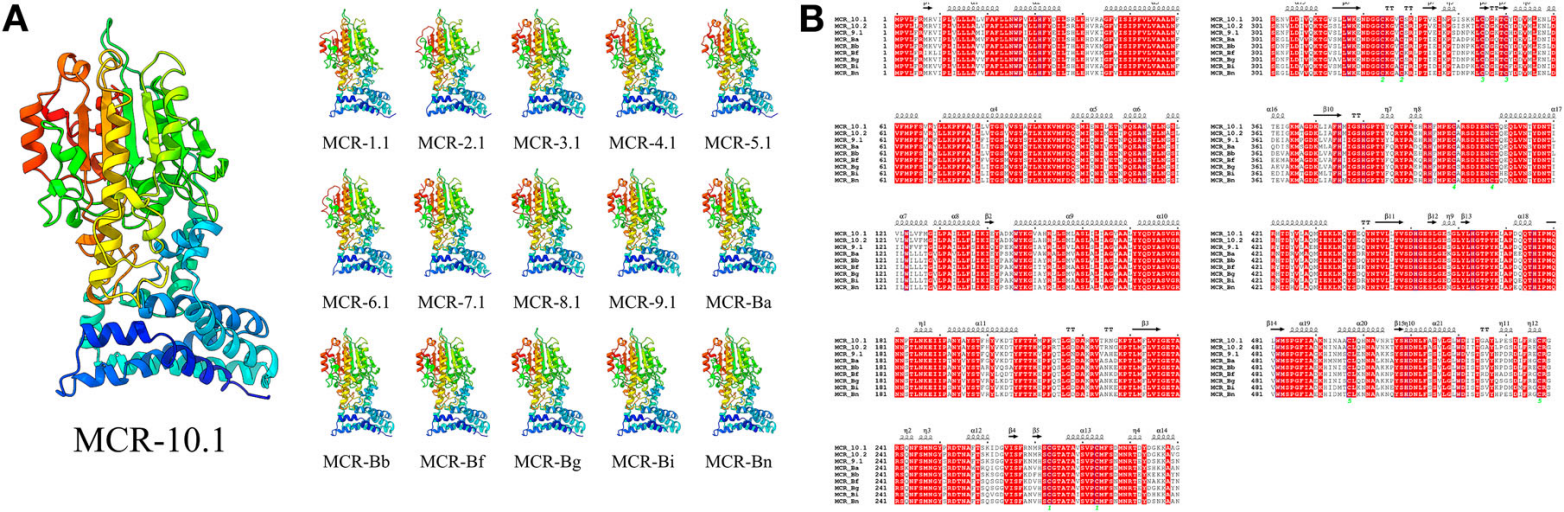
Structures of MCR-10, other reported MCR proteins (MCR-1 to -9) and MCR-B of various *Buttiauxella* species. (A) Structural models were constructed using Phyre2 [27] and show the transmembrane-anchored and soluble periplasmic domains of the phosphoethanolamine transferase. (B) Secondary structures of MCR-10, MCR-9 and MCR-B. The alignment of amino acid sequences and the prediction of secondary structures were performed using ESPript 3 [29]. Secondary structure elements, αhelixes, β sheets, and 3_10_-helixes (representing by η), are indicated. β-strands are rendered as arrows, and strict β-turns are shown as TT letters.

Three-dimensional (3D) models showed that both the membrane-anchored domain and the soluble catalytic domain of MCR-1 to -10 and MCR-Bs had high levels of conservation (Figure 2(A)). The N-terminal membrane-anchored domain and the C-terminal soluble catalytic domain of these MCR proteins were conserved in both amino acids and structural elements (Figure 2(B)).

### The mobilization of *mcr-10* may be due to site-specific recombination

**Figure 3.**
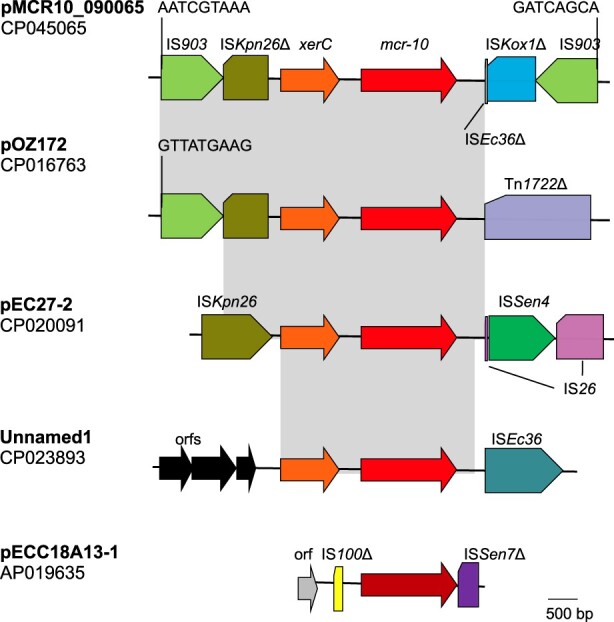
Genetic contexts of *mcr-10*. Gene *xerC* (shown in orange) encodes a XerC-type tyrosine recombinase, which may mediate mobilization of adjacent genetic components via site-specific recombination. Δ represents truncated insertion sequences or transposons. Identical regions are highlighted by grey rectangles. On pMCR10_090065, two copies of IS*903* are located at upstream and downstream of *xerC-mcr-10* (*mcr-10* is shown in red) and the 9-bp abutting sequences are indicated. On pOZ171, there is an IS*903* at upstream with the 9-bp abutting sequence being shown. However, there is no IS*903* at downstream of *xerC-mcr-10* but instead, transposon Tn*1722* is presented. On pEC27-2, there is no IS*903*. A complete IS*Kpn26* is present at upstream of *xerC-mcr-10* and an IS*26*, which is interrupted by the insertion of IS*Sen4*, is at downstream. On an unnamed plasmid (accession no. CP023893), several open reading frames (orfs) without known function are present at upstream of *xerC-mcr-10*, while IS*Ec36* is present at downstream. On pECC18A13-1, the *mcr-10* encodes an MCR-10 variant with 15 amino acids different from MCR-10 encoded by the other plasmids and is shown in dark red. No *xerC* is present, while truncated IS*100* and truncated IS*Sen7* are located at upstream and downstream of the *mcr-10*, respectively.

On pMCR10_090065 and three other plasmids carrying *mcr-10*, *mcr-10* was located at the immediate downstream of a XerC-type tyrosine recombinase-encoding gene, designated *xerC* here (Figure 3). It has been known that XerC-type tyrosine recombinases are able to mediate mobilization of adjacent genetic components including antimicrobial resistance genes via site-specific recombination [33,34]. In *Enterobacter* spp., an XerC-type tyrosine recombinase has been identified to mediate the mobilization of *bla*_IMI_, a carbapenemase gene [35]. Therefore, the mobilization of *mcr-10* may also be mediated by the XerC-type tyrosine recombinase. As the downstream sequence of *mcr-10* was truncated by insertion sequence IS*Ec36*, it is therefore impossible to identify the recombination sites recognized by the XerC-type tyrosine recombinase. Of note, two copies of IS*903* were located at upstream and downstream of *xerC-mcr-10* on pMCR10_090065, respectively, and could form a composite transposon. On insertion, IS*903* generates 9-bp direct target repeats but the 9-bp sequences abutting the two IS*903* were different, suggesting that the region bracketed by IS*903* was not due to direct insertion. Nonetheless, the IS*903­*-formed composite transposon has the potential to mediate mobilization of the intervening genetic components. In contrast, IS*903* has also been found upstream of *mcr-9* but other insertion sequences such as IS*26* are present downstream instead [32].

## Discussion

*Enterobacteriaceae* is the most common pathogen causing human infections [36]. Multi-drug resistant organisms such as carbapenem-resistant *Enterobacteriaceae* (CRE) have become a major global challenge [37]. Colistin is a last resort antimicrobial agent and one of the only options to treat serious infections caused by CRE. However, *Enterobacteriaceae* strains with acquired colistin resistance have emerged worldwide, which are significantly jeopardizing the efficacy of colistin [38]. Plasmid-borne *mcr* colistin resistance genes can be transmitted across *Enterobacteriaceae* species and may have spread to multiple continents, representing a particular threat to public health and clinical management in the whole world [39]. Monitoring the spread antimicrobial resistance is a core component of strategies for combating antimicrobial resistance [40]. Identification of new *mcr* genes can be used to improve the monitoring of plasmid-borne colistin resistance and may, therefore, help to develop effective control measures.

In this study, we identified a novel *mcr* gene in strain 090065 of *Enterobacter roggenkampii*. *mcr-10* has the highest nucleotide identity (79.69%) with *mcr-9* and encodes MCR-10 with 82.93% amino acids identical to MCR-9. *mcr-10* confers 4-fold increase in colistin MIC (from 1 to 4 mg/L) when cloned into a colistin-susceptible *E. roggenkampii* strain. This suggests that *mcr-10* was indeed a colistin resistance gene. Of note, strain 090065 carrying *mcr-10* was susceptible to colistin (MIC, 2 mg/L), while the originally colistin-susceptible *E. roggenkampii* strain became resistant to colistin (MIC, 4 mg/L) after receiving *mcr-10* by cloning. The discrepancy in colistin susceptibility is likely due to the expression as *mcr-10* was carried by a large naturally occurring plasmid in strain 090065 but was cloned onto the small-size vector pBC SK in strain 120033. Nonetheless, such discrepancy warrants further studies. We revealed that *mcr-10* was adjacent to site-specific recombinase-encoding gene and was bracketed by IS*903* on an IncFIA plasmid in strain 090065, indicating that *mcr-10* has the potential to be mobile. We also found that *mcr-10* has been present in a few genera of the family *Enterobacteriaceae* with a global distribution. Of note, the earliest match of *mcr-10* in GenBank was plasmid pOZ172 of a *Citrobacter freundii* clinical strain, which was recovered in 1998 in Guangzhou, southern China, suggesting that *mcr-10* has been mobilized by plasmids within *Enterobacteriaceae* for decades. We showed that MCR-10, MCR-9 and MCR-B proteins originated from a common ancestor. The large number and diffuse distribution of amino acid variations between MCR-10 and MCR-B proteins suggest that MCR-10 is not directly derived from these *Buttiauxella* species but may have originated from a yet-to-identified species that are closely related to known *Buttiauxella* species.

In conclusion, we identified and characterized a novel *mcr* gene and we also found that the gene has been spread globally under the radar. This suggests that the novel *mcr* gene is of significance for health. Our findings are essential for developing effective countermeasures including surveillance.

## Supplementary Material

Supplemental Material

## References

[1] Li J, Nation RL, Turnidge JD, et al. Colistin: the re-emerging antibiotic for multidrug-resistant Gram-negative bacterial infections. Lancet Infect Dis. 2006;6:589–601. doi: 10.1016/S1473-3099(06)70580-1 16931410

[2] Liu Y-Y, Wang Y, Walsh TR, et al. Emergence of plasmid-mediated colistin resistance mechanism MCR-1 in animals and human beings in China: a microbiological and molecular biological study. Lancet Infect Dis. 2016;16:161–168. doi: 10.1016/S1473-3099(15)00424-7 26603172

[3] Xavier BB, Lammens C, Ruhal R, et al. Identification of a novel plasmid-mediated colistin-resistance gene, *mcr-2*, in *Escherichia coli*, Belgium. Euro Surveill. 2016 Jun;21:30280. doi: 10.2807/1560-7917.ES.2016.21.27.30280 27416987

[4] Yin W, Li H, Shen Y, et al. Novel plasmid-mediated colistin resistance gene *mcr-3* in *Escherichia coli*. mBio. 2017;8:e00543-17. doi: 10.1128/mBio.00543-17 28655818 PMC5487729

[5] Carattoli A, Villa L, Feudi C, et al. Novel plasmid-mediated colistin resistance *mcr-4* gene in *Salmonella* and *Escherichia coli*, Italy 2013, Spain and Belgium, 2015 to 2016. Euro Surveill. 2017;22:30589. doi: 10.2807/1560-7917.ES.2017.22.31.30589 28797329 PMC5553062

[6] Borowiak M, Fischer J, Hammerl JA, et al. Identification of a novel transposon-associated phosphoethanolamine transferase gene, *mcr-5*, conferring colistin resistance in D-tartrate fermenting *Salmonella enterica* subsp. *enterica* serovar Paratyphi B. J Antimicrob Chemother. 2017;72:3317–3324. doi: 10.1093/jac/dkx327 28962028

[7] AbuOun M, Stubberfield EJ, Duggett NA, et al. *mcr-1* and *mcr-2* variant genes identified in *Moraxella* species isolated from pigs in Great Britain from 2014 to 2015. J Antimicrob Chemother. 2017;72:2745–2749. doi: 10.1093/jac/dkx286 29091227 PMC5890717

[8] Yang YQ, Li YX, Lei CW, et al. Novel plasmid-mediated colistin resistance gene *mcr-7.1* in *Klebsiella pneumoniae*. J Antimicrob Chemother. 2018;73:1791–1795. doi: 10.1093/jac/dky111 29912417

[9] Wang X, Wang Y, Zhou Y, et al. Emergence of a novel mobile colistin resistance gene, *mcr-8*, in NDM-producing *Klebsiella pneumoniae*. Emerg Microbes Infect. 2018;7;122. 29970891 10.1038/s41426-018-0124-z PMC6030107

[10] Carroll LM, Gaballa A, Guldimann C, et al. Identification of novel mobilized colistin resistance gene *mcr-9* in a multidrug-resistant, colistin-susceptible *Salmonella enterica* serotype Typhimurium isolate. mBio. 2019;10:00853–19. doi: 10.1128/mBio.00853-19 PMC6509194 31064835

[11] Martins-Sorenson N, Snesrud E, Xavier DE, et al. A novel plasmid-encoded *mcr-4.3* gene in a colistin-resistant *Acinetobacter baumannii* clinical strain. J Antimicrob Chemother. 2019;75:60–64. doi: 10.1093/jac/dkz413 PMC6910164 31578567

[12] Caselli E, D'Accolti M, Soffritti I, et al. Spread of *mcr-1*-driven colistin resistance on hospital surfaces, Italy. Emerg Infect Dis. 2018;24:1752–1753. doi: 10.3201/eid2409.171386 30124425 PMC6106434

[13] Sun J, Zhang H, Liu YH, et al. Towards understanding MCR-like colistin resistance. Trends Microbiol. 2018;26:794–808. doi: 10.1016/j.tim.2018.02.006 29525421

[14] CLSI. Performance standards for antimicrobial susceptibility testing; twenty-seventh informational supplement. M100-S27. Wayne (PA): Clinical and Laboratory Standards Institute; 2017.

[15] Bankevich A, Nurk S, Antipov D, et al. SPAdes: a new genome assembly algorithm and its applications to single-cell sequencing. J Comput Biol. 2012;19:455–477. doi: 10.1089/cmb.2012.0021 22506599 PMC3342519

[16] Wick RR, Judd LM, Gorrie CL, et al. Unicycler: resolving bacterial genome assemblies from short and long sequencing reads. PLoS Comput Biol. 2017;13:e1005595. doi: 10.1371/journal.pcbi.1005595 28594827 PMC5481147

[17] Walker BJ, Abeel T, Shea T, et al. Pilon: an integrated tool for comprehensive microbial variant detection and genome assembly improvement. PLoS One. 2014;9:e112963. doi: 10.1371/journal.pone.0112963 25409509 PMC4237348

[18] Seemann T. Prokka: rapid prokaryotic genome annotation. Bioinformatics. 2014;30:2068–2069. doi: 10.1093/bioinformatics/btu153 24642063

[19] Richter M, Rossello-Mora R, Glockner F O, et al. JSpeciesWS: a web server for prokaryotic species circumscription based on pairwise genome comparison. Bioinformatics. 2016;32:929–931. doi: 10.1093/bioinformatics/btv681 26576653 PMC5939971

[20] Richter M, Rossello-Mora R. Shifting the genomic gold standard for the prokaryotic species definition. Proc Natl Acad Sci U S A. 2009;106:19126–19131. doi: 10.1073/pnas.0906412106 19855009 PMC2776425

[21] Dvorak P, Chrast L, Nikel PI, et al. Exacerbation of substrate toxicity by IPTG in *Escherichia coli* BL21(DE3) carrying a synthetic metabolic pathway. Microb Cell Fact. 2015;14:201. doi: 10.1186/s12934-015-0393-3 26691337 PMC4687329

[22] Coque TM, Oliver A, Perez-Diaz JC, et al. Genes encoding TEM-4, SHV-2, and CTX-M-10 extended-spectrum β-lactamases are carried by multiple *Klebsiella pneumoniae* clones in a single hospital (Madrid, 1989 to 2000). Antimicrob Agents Chemother. 2002;46:500–510. doi: 10.1128/AAC.46.2.500-510.2002 11796363 PMC127031

[23] Bio-Rad Laboratories I. Gene Pulser Xcell electroporation system instruction manual. Alfred Nobel Drive, CA. 2000.

[24] Loytynoja A. Phylogeny-aware alignment with PRANK. Methods Mol Biol. 2014;1079:155–170. doi: 10.1007/978-1-62703-646-7_10 24170401

[25] Stamatakis A. RAxML version 8: a tool for phylogenetic analysis and post-analysis of large phylogenies. Bioinformatics. 2014;30:1312–1313. doi: 10.1093/bioinformatics/btu033 24451623 PMC3998144

[26] Anandan A, Evans GL, Condic-Jurkic K, et al. Structure of a lipid A phosphoethanolamine transferase suggests how conformational changes govern substrate binding. Proc Natl Acad Sci USA. 2017;114:2218–2223. doi: 10.1073/pnas.1612927114 28193899 PMC5338521

[27] Kelley LA, Mezulis S, Yates CM, et al. The Phyre2 web portal for protein modeling, prediction and analysis. Nat Protoc. 2015;10:845–858. doi: 10.1038/nprot.2015.053 25950237 PMC5298202

[28] Pettersen EF, Goddard TD, Huang CC, et al. UCSF Chimera – a visualization system for exploratory research and analysis. J Comput Chem. 2004;25:1605–1612. doi: 10.1002/jcc.20084 15264254

[29] Robert X, Gouet P. Deciphering key features in protein structures with the new ENDscript server. Nucleic Acids Res. 2014;42:W320–W324. doi: 10.1093/nar/gku316 24753421 PMC4086106

[30] Sutton GG, Brinkac LM, Clarke TH, et al. *Enterobacter hormaechei* subsp. *hoffmannii* subsp. nov., *Enterobacter hormaechei* subsp. *xiangfangensis* comb. nov., *Enterobacter roggenkampii* sp. nov., and *Enterobacter muelleri* is a later heterotypic synonym of *Enterobacter asburiae* based on computational analysis of sequenced *Enterobacter* genomes. F1000Res. 2018;7:521. doi: 10.12688/f1000research.14566.1 30430006 PMC6097438

[31] Partridge SR, Di Pilato V, Doi Y, et al. Proposal for assignment of allele numbers for mobile colistin resistance (*mcr*) genes. J Antimicrob Chemother. 2018;73:2625–2630. doi: 10.1093/jac/dky262 30053115 PMC6148208

[32] Kieffer N, Royer G, Decousser JW, et al. *. mcr-9*, an inducible gene encoding an acquired phosphoethanolamine transferase in *Escherichia coli*, and its origin. Antimicrob Agents Chemother. 2019;63:e00965-19. doi: 10.1128/AAC.00965-19 31209009 PMC6709461

[33] Castillo F, Benmohamed A, Szatmari G. Xer site specific recombination: double and single recombinase systems. Front Microbiol. 2017;8:453. 28373867 10.3389/fmicb.2017.00453 PMC5357621

[34] Midonet C, Barre FX. Xer site-specific recombination: promoting vertical and horizontal transmission of genetic information. Microbiol Spectr. 2014;2. DOI:10.1128/microbiolspec.MDNA3-0056-2014. 26104463

[35] Antonelli A, D'Andrea MM, Di Pilato V, et al. Characterization of a novel putative Xer-dependent integrative mobile element carrying the *bla*_NMC-A_ carbapenemase gene, inserted into the chromosome of members of the *Enterobacter cloacae* complex. Antimicrob Agents Chemother. 2015;59:6620–6624. doi: 10.1128/AAC.01452-15 26248383 PMC4576029

[36] Adeolu M, Alnajar S, Naushad S, et al. Genome-based phylogeny and taxonomy of the ‘*enterobacteriales*': proposal for *Enterobacterales* ord. nov. divided into the families *Enterobacteriaceae*, *Erwiniaceae* fam. nov., *Pectobacteriaceae* fam. nov., *Yersiniaceae* fam. nov., *Hafniaceae* fam. nov., *Morganellaceae* fam. nov., and *Budviciaceae* fam. nov. Int J Syst Evol Microbiol. 2016;66:5575–5599. doi: 10.1099/ijsem.0.001485 27620848

[37] Nordmann P, Naas T, Poirel L. Global spread of carbapenemase-producing Enterobacteriaceae. Emerg Infect Dis. 2011;17:1791–1798. doi: 10.3201/eid1710.110655 22000347 PMC3310682

[38] Tzouvelekis LS, Markogiannakis A, Psichogiou M, et al. Carbapenemases in *Klebsiella pneumoniae* and other Enterobacteriaceae: an evolving crisis of global dimensions. Clin Microbiol Rev. 2012;25:682–707. doi: 10.1128/CMR.05035-11 23034326 PMC3485753

[39] Poirel L, Jayol A, Nordmann P. Polymyxins: antibacterial activity, susceptibility testing, and resistance mechanisms encoded by plasmids or chromosomes. Clin Microbiol Rev. 2017;30:557–596. doi: 10.1128/CMR.00064-16 28275006 PMC5355641

[40] CDC. Facility guidance for control of carbapenem-resistant Enterobacteriaceae (CRE). Atlanta (GA): CDC; 2015 Nov.

