# Supplementary material for: Identification of novel mobile colistin resistance gene mcr-10
Source: Emerg Microbes Infect. 2020 Mar 2;9(1):508–16. doi: 10.1080/22221751.2020.1732231 (PMC7067168; doi:10.1080/22221751.2020.1732231)
Supplement: Supplemental Material [file TEMI_A_1732231_SM6243.docx]

**Supplementary Files**

**Fig. S1. Alignment of the MCR-10 variant in *Enterobacter* strain 18A13 with the MCR-10 identified in the present study.** Query, MCR-10 in strain 090065; Sbjct, subject, the MCR-10 variant in *Enterobacter* strain 18A13.

Query 1 MPVLFRMRVIPLVLLLALVFAFLLNWPVLLHFYDILSRLEHVRAGFVISIPFVLVAALNF 60

MPVL RMRVIPLVLLLALVFAFLLNWPVLLHFYDILSRLEHVRAGFVISIPFVLVAALNF

Sbjct 1 MPVLLRMRVIPLVLLLALVFAFLLNWPVLLHFYDILSRLEHVRAGFVISIPFVLVAALNF 60

Query 61 VFMPFSVRYLLKPFFALLLVTGSVVSYATLKYKVMFDQSMIENILETNPQEAHAYLNGSL 120

VFMPFSVRYLLKPFFALLLVTGSVVSYATLKYKVMFDQSMIENI+ET PQEAH+YLN SL

Sbjct 61 VFMPFSVRYLLKPFFALLLVTGSVVSYATLKYKVMFDQSMIENIMETTPQEAHSYLNASL 120

Query 121 VLWLVFMGILPAILLFLIKIEYADKWYKGVAHRLLSMLASLILIAGVAALYYQDYASVGR 180

VLWLVFMGILPAILLFLIKIEYADKWYKG+AHRLLSMLASL LIAGVAALYYQDYASVGR

Sbjct 121 VLWLVFMGILPAILLFLIKIEYADKWYKGLAHRLLSMLASLALIAGVAALYYQDYASVGR 180

Query 181 NNPTLNKEIIPANYAYSTFHYVKDTYFTTKMPFRTLGDDARRVTRNGKPTLMFLVIGETA 240

NNPTLNKEIIPANYAYSTFHYVKDTYFTTKMPFRTLGDDA+RVTRNGKPTLMFLVIGETA

Sbjct 181 NNPTLNKEIIPANYAYSTFHYVKDTYFTTKMPFRTLGDDAKRVTRNGKPTLMFLVIGETA 240

Query 241 RSQNFSMNGYPRDTNAFTSKIDGVISFRNMRSCGTATAVSVPCMFSDMNRTDYDGKKAAG 300

RSQNFSMNGYPRDTNAFTSKIDGVISFRNMRSCGTATAVSVPCMFSDMNRTDYDGKKA G

Sbjct 241 RSQNFSMNGYPRDTNAFTSKIDGVISFRNMRSCGTATAVSVPCMFSDMNRTDYDGKKAVG 300

Query 301 SENVLDIVQKTGVSLLWKENDGGCKGVCSRIPTVEINPGISKKLCDGKTCYDDVMLENLD 360

SENVLDIVQKTG SLLWKENDGGCKGVCSRIPTVEINPGISK LCDGKTCYDDVMLENLD

Sbjct 301 SENVLDIVQKTGGSLLWKENDGGCKGVCSRIPTVEINPGISKTLCDGKTCYDDVMLENLD 360

Query 361 TEIGKMAGDKLIAFHMIGSHGPTYYQRYPAEHRHFMPECARSDIENCTQEQLVNTYDNTI 420

EIGKM GDKLIAFHMIGSHGPTYYQRYPAEHRHFMPECARSDIENCTQEQLVNTYDNTI

Sbjct 361 TEIGKMTGDKLIAFHMIGSHGPTYYQRYPAEHRHFMPECARSDIENCTQEQLVNTYDNTI 420

Query 421 RHTDYVLAQMIEKLKQYSEQYNTVLLYVSDHGESLGESGLYLHGTPYKLAPDQQTHIPMQ 480

RHTDYVLAQMIEKLKQYSEQYNTVLLYVSDHGESLGESGLYLHGTPYKLAPDQQTHIPMQ

Sbjct 421 RHTDYVLAQMIEKLKQYSEQYNTVLLYVSDHGESLGESGLYLHGTPYKLAPDQQTHIPMQ 480

Query 481 LWMSPGFIAAKNINAACLQHNAVNRTYSHDNLFASVLGLWDITTGAYLPESDLFRECRG 539

LWMSPGFIA KNINAACLQHNAVN+TYSHDNLFASVLGLWDITTGAYLP SDLFRECRG

Sbjct 481 LWMSPGFIADKNINAACLQHNAVNKTYSHDNLFASVLGLWDITTGAYLPGSDLFRECRG 539

**Fig. S2. The maximum likelihood phylogeny of *mcr* genes and *mcr*-like ones from *Buttiauxella* species.** The nucleotide sequences of *mcr* genes were retrieved from Bacterial Antimicrobial Resistance Reference Gene Database (BioProject no. PRJNA313047). The *mcr*-like genes of species from genus *Buttiauxella* were retrieved from their whole genome assemblies. Along with the two alleles of *mcr-10* found in this study, the amino acid sequences of all genes (n=75) were aligned using Prank v1.70427 (20) with 50 iterations first, followed by aligning corresponding nucleotide sequences using aligned amino acid sequences as the guide in the same program. The aligned nucleotide sequences were fed into RAxML v8.2.12 (21) with a 1,000-bootstrap test under the GTRGAMMA model for inferring phylogenies. The tree is middle-point rooted and the blue and green strips separate different *mcr* families with *mcr-10* identified in the present study being highlighted in red. Bootstrap results are indicated by colour gradient on the branches, starting from 50% shown as red and up to 100% shown as green. *mcr*-like genes in *Buttiauxella* species are named here according to the species. *mcr-Ba*, *mcr-Bb*, *mcr-Bf*, *mcr-Bg*, *mcr-Bi*, and *mcr-Bn* are *mcr*-like genes from *Buttiauxella agrestis* strain ATCC 33320^T^ (accession no. JMPI00000000), *Buttiauxella brennerae* ATCC 51605^T^ (accession no. LXER00000000), *Buttiauxella ferragutiae* ATCC 51602^T^ (accession no. LXEQ00000000), *Buttiauxella gaviniae* ATCC 51604^T^ (accession no. LXEP00000000), *Buttiauxella izardii* CCUG35510^T^ (accession no. QZWH01000000), and *Buttiauxella noackiae* ATCC 51607^T^ (accession no. LXEO00000000), respectively.
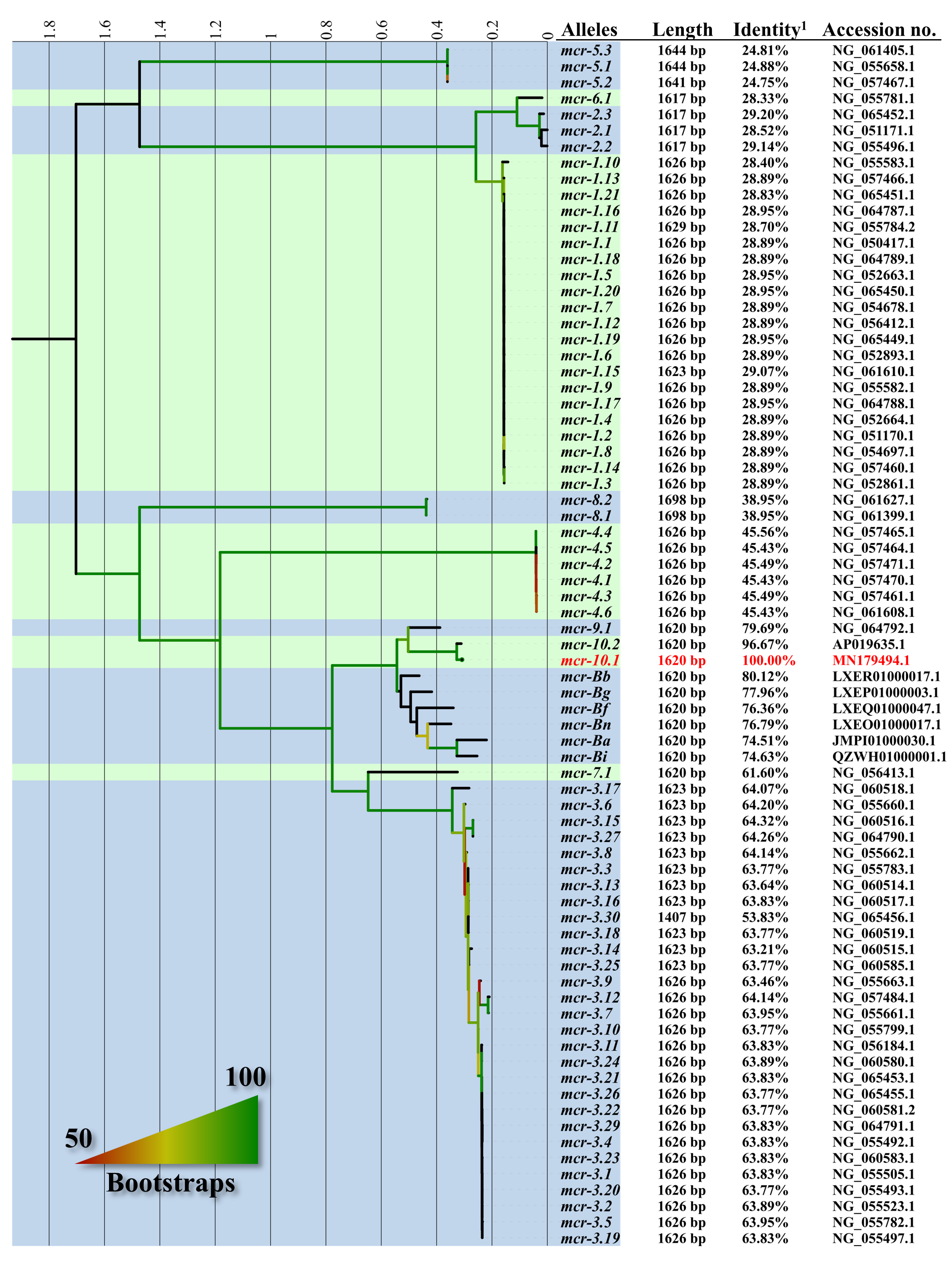


**Fig. S3. Alignment of MCR-10 of strain 090065 with MCR-B of *Buttiauxella* species and MCR-9.** MCR-10.1 refers to MCR-10 in strain 090065, while MCR-10.2 refers to the MCR-10 variant in strain *Enterobacter* strain 18A13. MCR-Ba, MCR-Bb, MCR-Bf, MCR-Bg, MCR-Bi, and MCR-Bn are MCR-like proteins from *Buttiauxella agrestis* strain ATCC 33320^T^ (accession no. JMPI00000000), *Buttiauxella brennerae* ATCC 51605^T^ (accession no. LXER00000000), *Buttiauxella ferragutiae* ATCC 51602^T^ (accession no. LXEQ00000000), *Buttiauxella gaviniae* ATCC 51604^T^ (accession no. LXEP00000000), *Buttiauxella izardii* CCUG35510^T^ (accession no. QZWH01000000), and *Buttiauxella noackiae* ATCC 51607^T^ (accession no. LXEO00000000), respectively.

MCR-10.1 MPVLFRMRVIPLVLLLALVFAFLLNWPVLLHFYDILSRLEHVRAGFVISIPFVLVAALNF 60

MCR-10.2 MPVLLRMRVIPLVLLLALVFAFLLNWPVLLHFYDILSRLEHVRAGFVISIPFVLVAALNF 60

MCR-9.1 MPVLFRVKVIPLVLLLAMIFAFLLNWPILLHFYEILSHLEHVKIGFVISIPFVLVAALNV 60

MCR-Ba MPVLFRMKVIPLVLLLAMVFAFLLNWPVLLHFYDILSHLEHDKAGFVISIPFVLVAALNF 60

MCR-Bb MPVLFRMKVVPLILLLAVVFAFLLNWPVLLHFYDILTHLERVKMGFVISIPFVLVAALNF 60

MCR-Bf MPVLFRIKLIPLVLLLAVVFAFLLNWPVLLHFYDILTHLEHVKMGFAISIPFVLVAALNF 60

MCR-Bg MPVLFRMKVIPLILLLAVVFAFLLNWPVLLHFYDILTHLEHVKIGFVISIPFVLVAALNF 60

MCR-Bi MPVLFRMKVVPLVLLLAVVFAFLLNWPVLLHFYDILSHLEHVKAGFVISIPFVLVAALNF 60

MCR-Bn MPVLFRMKVIPLILLLAVVFAFLLNWPVLLHFYNILTHLEHVKIGFVISIPFVLVAALNF 60

**** * ** **** ******** ***** ** ** ** ************

MCR-10.1 VFMPFSVRYLLKPFFALLLVTGSVVSYATLKYKVMFDQSMIENILETNPQEAHAYLNGSL 120

MCR-10.2 VFMPFSVRYLLKPFFALLLVTGSVVSYATLKYKVMFDQSMIENIMETTPQEAHSYLNASL 120

MCR-9.1 VFMPFSVRFLLKPFFALLFITGSLVSYSTLKYKVMFDQTMIQNIIETNPQEAHSYLNGSI 120

MCR-Ba VFMPFSVRYLLKPFFALLLITGSMVSYSTLKYKVMFDQSMIQNIVETNPQEAHSYLNGSI 120

MCR-Bb VFMPFSIRYLLKPFFALLLVTGSMVSYSTLKYKVMFDQSMIENIIETNPQEAHSYLNGSI 120

MCR-Bf VFMPFSIRFLLKPFFALLFVTGSMVSYSTLKYKVMFDQSMIQNIVETNPQEAHSYLNGSI 120

MCR-Bg VFMPFSIRFLLKPFFAFLLITGSMVSYATLKYKVMFDQSMIENIIETNPQEAHSYLNGSI 120

MCR-Bi VFMPFSIRFLLKPFFALLLITGSMVSYSTLKYKVMFDQSMIQNIVETNPQEAHSYLNGSI 120

MCR-Bn VFMPFSIRFLLKPFFALLLITGSMVSYSTLKYKVMFDQSMIQNIIETNPQEAHSYLNGSI 120

****** * ******* * *** *** ********** ** ** ** ***** *** *

MCR-10.1 VLWLVFMGILPAILLFLIKIEYADKWYKGVAHRLLSMLASLILIAGVAALYYQDYASVGR 180

MCR-10.2 VLWLVFMGILPAILLFLIKIEYADKWYKGLAHRLLSMLASLALIAGVAALYYQDYASVGR 180

MCR-9.1 IIWFVFTGILPAILLFSIKIQYPEKWYKGIAYRLLSVLASLSLIAGVAALYYQDYASVGR 180

MCR-Ba ILWLLLTGVLPAVLLFLIKIEYPSKWYKGVAWRALSMVASLALIAGVAAIYYQDYASVGR 180

MCR-Bb ILWILLTGVLPAILLFLIKIEYPAKWYKGIAYRLLSMVASLVLIAGIAALYYQDYASVGR 180

MCR-Bf ILWLLLTGILPAILLFLIKIEYPAKWYKGITYRLLSMAASLLLIAGVAALYYQDYASVGR 180

MCR-Bg ILWILLTGVLPAILLFLIKIEYPARWYKGIAYRLLSMVASLALIAGVAALYYQDYASVGR 180

MCR-Bi ILWLLLTGVLPAILLFLIKIEYPAKWYKGIAYRLLSMAASLVLIAGVAALYYQDYASVGR 180

MCR-Bn ILWILLTGVLPAILLFLIKIEYPSKWYKGIAYRLLSMLASLALVAGVAALYYQDYASVGR 180

* * *** *** *** * **** * ** *** * ** ** **********

MCR-10.1 NNPTLNKEIIPANYAYSTFHYVKDTYFTTKMPFRTLGDDARRVTRNGKPTLMFLVIGETA 240

MCR-10.2 NNPTLNKEIIPANYAYSTFHYVKDTYFTTKMPFRTLGDDAKRVTRNGKPTLMFLVIGETA 240

MCR-9.1 NNSTLNKEIIPANYAYSTFQYVKDTYFTTKVPFQTLGNDAKRVVAHEKPTLMFLVIGETA 240

MCR-Ba NNSTLNKEIIPTNYVYSTFGYVKDTYFTTKEPFQTLGDDAKRVASADKPTLMFLVIGETA 240

MCR-Bb NNSTLNKEIIPANYVYSTARYVQSAYFTTKEPFQSLGDDAKRVANKEKPTLMFLVIGETA 240

MCR-Bf NNSTLNKEIIPANYVYSTVRYLQDTYFTTKEPFQTLGDDAKRVANKDKPTLMFLVIGETA 240

MCR-Bg NNSTLNKEIIPANYVYSTVRYLKDTYFTTKEPFQSLGDDAKRVANKEKPTLMFLVIGETA 240

MCR-Bi NNSTLNKEIIPSNYVYSTFGYVKDTYFTTKEPFQTLGDDAKRVANKEKPTLMILVIGETA 240

MCR-Bn NNSTLNKEIIPANYVYSTVRYLKDTYFTTKEPFQTLGDDAIRVANKEKPTLMFLVIGETA 240

** ******** ** *** * ***** ** ** ** ** ***** *******

MCR-10.1 RSQNFSMNGYPRDTNAFTSKIDGVISFRNMRSCGTATAVSVPCMFSDMNRTDYDGKKAAG 300

MCR-10.2 RSQNFSMNGYPRDTNAFTSKIDGVISFRNMRSCGTATAVSVPCMFSDMNRTDYDGKKAVG 300

MCR-9.1 RSQNFSMNGYSRDTNAFTSKSGGVISFKNMHSCGTATAISVPCMFSNMNRTEYDSKKASN 300

MCR-Ba RSKNFSMNGYSRDTNAWTRQIGGVISFANVHSCGTATAVSVPCMFSDMNRTEYSHKRAAN 300

MCR-Bb RSQNFSMNGYSRDTNAFTSKSGGVISFKDFHSCGTATAVSVPCMFSDMNRTEYKEKKAYN 300

MCR-Bf RSQNFSMNGYDRDTNAFTSQSGDVISFKDVHSCGTATAVSVPCMFSNMNRTEYNAKKATN 300

MCR-Bg RSQNFSMNGYSRDTNAFTSQSGGVISFKDVHSCGTATAVSVPCMFSNMNRTEYDGKKAYN 300

MCR-Bi RSQNFSMNGYPRDTNAFTSQVGDVISFANVHSCGTATAISVPCMFSDMNRTDYDHKKAAN 300

MCR-Bn RSQNFSMNGYSRDTNAFTSQSGGVISFANVHSCGTATAVSVPCMFSNMNRTDYDSNKAAN 300

** ******* ***** * **** ******* ******* **** * *

MCR-10.1 SENVLDIVQKTGVSLLWKENDGGCKGVCSRIPTVEINPGISKKLCDGKTCYDDVMLENLD 360

MCR-10.2 SENVLDIVQKTGGSLLWKENDGGCKGVCSRIPTVEINPGISKTLCDGKTCYDDVMLENLD 360

MCR-9.1 SENFLDIVQKTGVSLLWKENDGGCKGVCSRIPTVEIKPSDNPKLCDGKTCHDEVMLENLD 360

MCR-Ba SEGLLDVVRKTGVSMLWKDNDGGCKGACTRIPTIEIKPTADPKLCDGQTCYDEVLLDNID 360

MCR-Bb SDNFLDIVQKTGVSLLWKENDGGCKGVCSRIPTIEIKPTDNPKLCDGETCYDDVMLESLD 360

MCR-Bf SENFLDIVQKTGVSLLWKDNDGGCKGVCKRLPTIEIKPTDNPKLCNGETCFDEVMLENID 360

MCR-Bg SDNFLDIVHKTGVAVLWKENDGGCKGVCSRIPTIEIKPTDNPKLCDGKTCYDDVMLENLD 360

MCR-Bi SEGLLDVVQKTGVSMLWKDNDGGCKGACTRIPTIDIKPTADPKLCDGETCFDEVMLDNID 360

MCR-Bn SEGLLDVVQKTGVSLLWKENDGGCKGACKRIPTIEIKPTDNPKLCDGETCYDDVMLENLD 360

* ** * *** *** ******* * * ** * * ** * ** * * * *

MCR-10.1 TEIGKMAGDKLIAFHMIGSHGPTYYQRYPAEHRHFMPECARSDIENCTQEQLVNTYDNTI 420

MCR-10.2 TEIGKMTGDKLIAFHMIGSHGPTYYQRYPAEHRHFMPECARSDIENCTQEQLVNTYDNTI 420

MCR-9.1 DEIAKMPGDKLVAFHIIGSHGPTYYLRYPAEHRHFMPECARSDIENCTQEQLVNTYDNTL 420

MCR-Ba QDISKMSGDKMLAFHLIGSHGPTYFQRYPADQRHYMPECARSDIENCTQEQLVNSYDNTI 420

MCR-Bb DEVAKMAGDKLVAFHMIGSHGPTYYQRYPAEHRHFMPECARSDIENCTQEQLVNTYDNTL 420

MCR-Bf EEMAKMAGDKLVAFHIIGSHGPTYFQRYPAKQRHFMPECARSDIENCTQEQLVNTYDNTI 420

MCR-Bg AEVAKMAGDKLVAFHMIGSHGPTYYQRYPAEHRHFMPECSRSDIENCTQEQLVNTYDNTI 420

MCR-Bi EDIAKMTGDKMLTFHLIGSHGPTYFQRYPADQRHYMPECARSDIENCTHEQLVNSYDNTI 420

MCR-Bn TEVAKMAGDKLIAFHMIGSHGPTYFQRYPAEHRHYMPECARSDIENCTQDQLVNTYDNTI 420

** *** ** ******** **** ** **** ******** **** ****

MCR-10.1 RHTDYVLAQMIEKLKQYSEQYNTVLLYVSDHGESLGESGLYLHGTPYKLAPDQQTHIPMQ 480

MCR-10.2 RHTDYVLAQMIEKLKQYSEQYNTVLLYVSDHGESLGESGLYLHGTPYKLAPDQQTHIPMQ 480

MCR-9.1 RYTDYVLAEMIEKLKNYSDQYNTVLLYVSDHGESLGESGLYLHGTPYKLAPDQQTHIPMQ 480

MCR-Ba RNTDRVMAQMIEKLKQYSDRYNTVLIYVSDHGESLGEKGLYLHGTPYKIAPEEQTHIPMQ 480

MCR-Bb RHTDYVLAQMIEKLKKYSDQYNTVLLYVSDHGESLGESGLYLHGTPYKLAPDQQTHIPMQ 480

MCR-Bf RYTDYVVAQMIEKLKLYSNKYNTVLLYVSDHGESLGESGLYLHGTPYKLAPDQQTHIPMQ 480

MCR-Bg RHTDYVLAQMIEKLKKYSDQYNTVLLYVSDHGESLGESGLYLHGTPYKLAPDQQTHIPMQ 480

MCR-Bi RNTDRVLAQTIEKLKQYSDRYNTVLLYVSDHGESLGEDGLYLHGTPYKIAPQEQTHIPMQ 480

MCR-Bn RHTDHVLAQMIEKLKQYSDRYNTVLLYVSDHGESLGENGLYLHGTPYKLAPEQQTHIPMQ 480

* ** * * ***** ** ***** *********** ********** ** *******

MCR-10.1 LWMSPGFIAAKNINAACLQHNAVNRTYSHDNLFASVLGLWDITTGAYLPESDLFRECRG 539

MCR-10.2 LWMSPGFIADKNINAACLQHNAVNKTYSHDNLFASVLGLWDITTGAYLPGSDLFRECRG 539

MCR-9.1 VWMSPGFIAGKHINMSCLENNAAKKSYSHDNLFSSILGLWDVSTSVYNPDRDLFRECRG 539

MCR-Ba VWMSPGFIAEKHMNMTCLQNNALKNAYSHDNLFSSVLGLWDISTSVYKPDMDIFHGCRS 539

MCR-Bb VWMSPGFIAEKHINISCLQDNAAKKPYSHDNLFSSVLGLWDISTSVYNPDSDLFRECRG 539

MCR-Bf VWMSPGFIADKHINMTCLKNNAAKNPYSHDNLFSSVLGIWDITTRDYHADSDLFRGCRS 539

MCR-Bg VWMSPGFIAEKHINISCLQDNAAKKPYSHDNLFSSVLGLWDISTSVYQSGSDLFRECRG 539

MCR-Bi VWMSPGFIADKHIDMTCLKNNALKNEYSHDNLFSSVLGLWDISTSVYKPDMDMFRGCRS 539

MCR-Bn VWMSPGFIAEKHIDMTCLKNNAAKNQYSHDNLFSSVLGLWDISTSVYHPESDIFRGCRS 539

******** * ** ** ******* * ** ** * * * * **
